# Supplementary material for: Modern anthropogenic drought in Central Brazil unprecedented during last 700 years
Source: Nat Commun. 2024 Feb 26;15:1728. doi: 10.1038/s41467-024-45469-8 (PMC11258244; doi:10.1038/s41467-024-45469-8)
Supplement: Supplementary file 1 — Supplementary Information [file 41467_2024_45469_MOESM1_ESM.pdf]

Supplementary Information

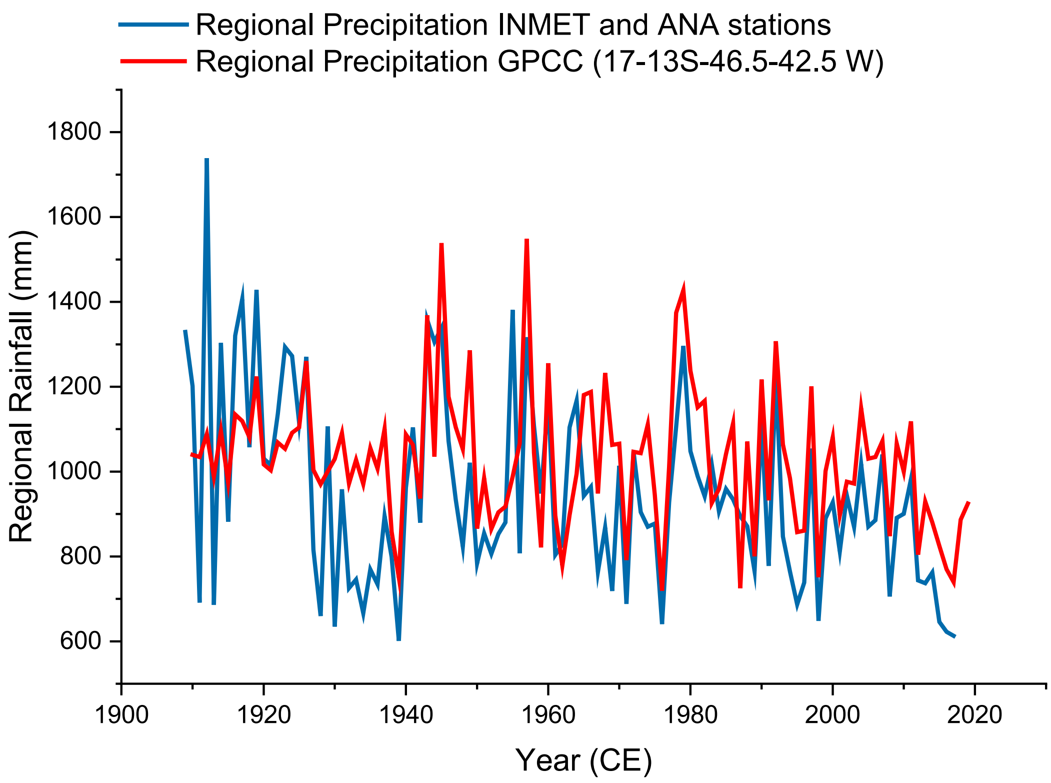

Figure S1 – Comparison between precipitation record from *Instituto Nacional de Meteorologia* (INMET) and *Agência Nacional de Água* (ANA) meteorological stations covering an area of ~ 4° × 4°, centered over the study site (see Table S1 for station locations) and regional precipitation from the Global Precipitation Climatology Centre (GPCC) dataset at 1° resolution (17° -13° S – 46.5° -42.5° W).

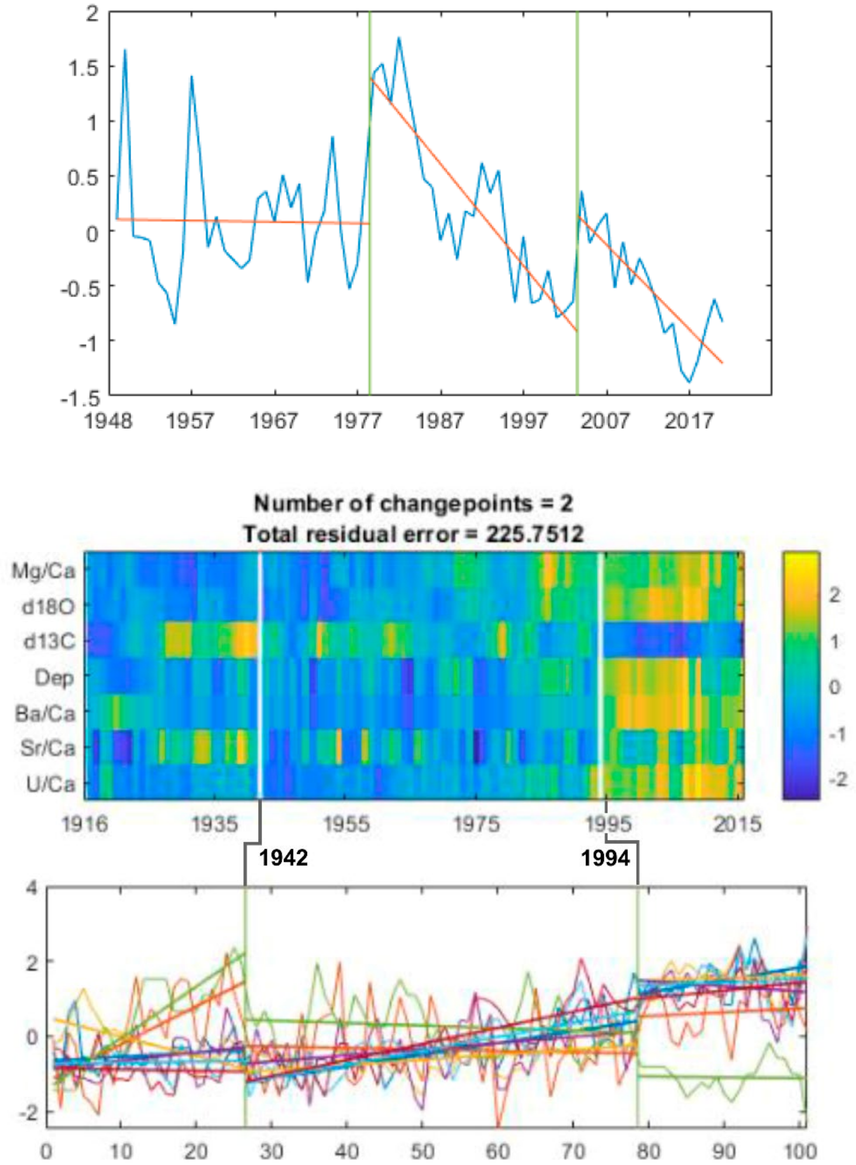

Figure S2 – Changepoint detection analysis using the algorithm[1]. Top panel: mean regional streamflow; middle panel: distribution of the z-score values of the speleothem proxies; bottom panel: change point detection analysis based on linear trend between proxy and depth ratio from Onça 2 speleothem covering the last 100 year from 1916 to 2016. Lines represent $\delta^{18}O$ (red); $\delta^{13}C$ (light blue); Mg/Ca (blue); Ba/Ca (yellow); Sr/Ca (purple); U/Ca (orange); deposition rate (green). Two change points in 1942 and 1994 are evident considering all the time series shown.

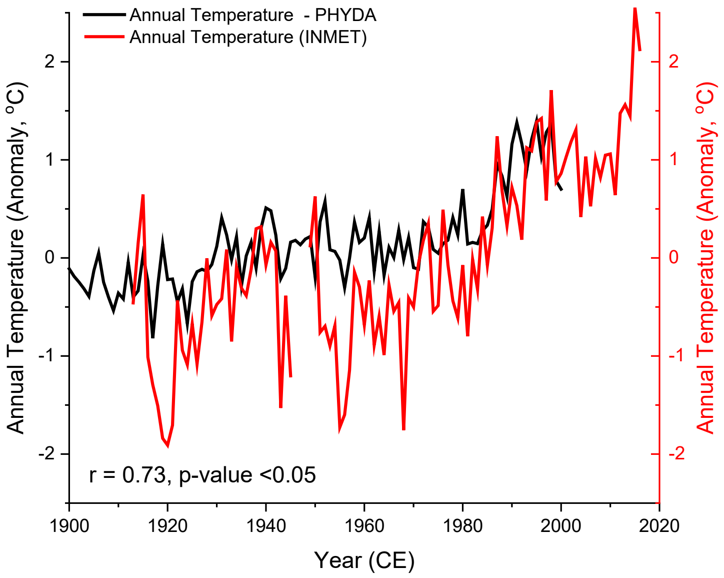

Figure S3 – Comparison between regional annual mean surface temperature anomaly from INMET station (Table S1) and PHYDA.

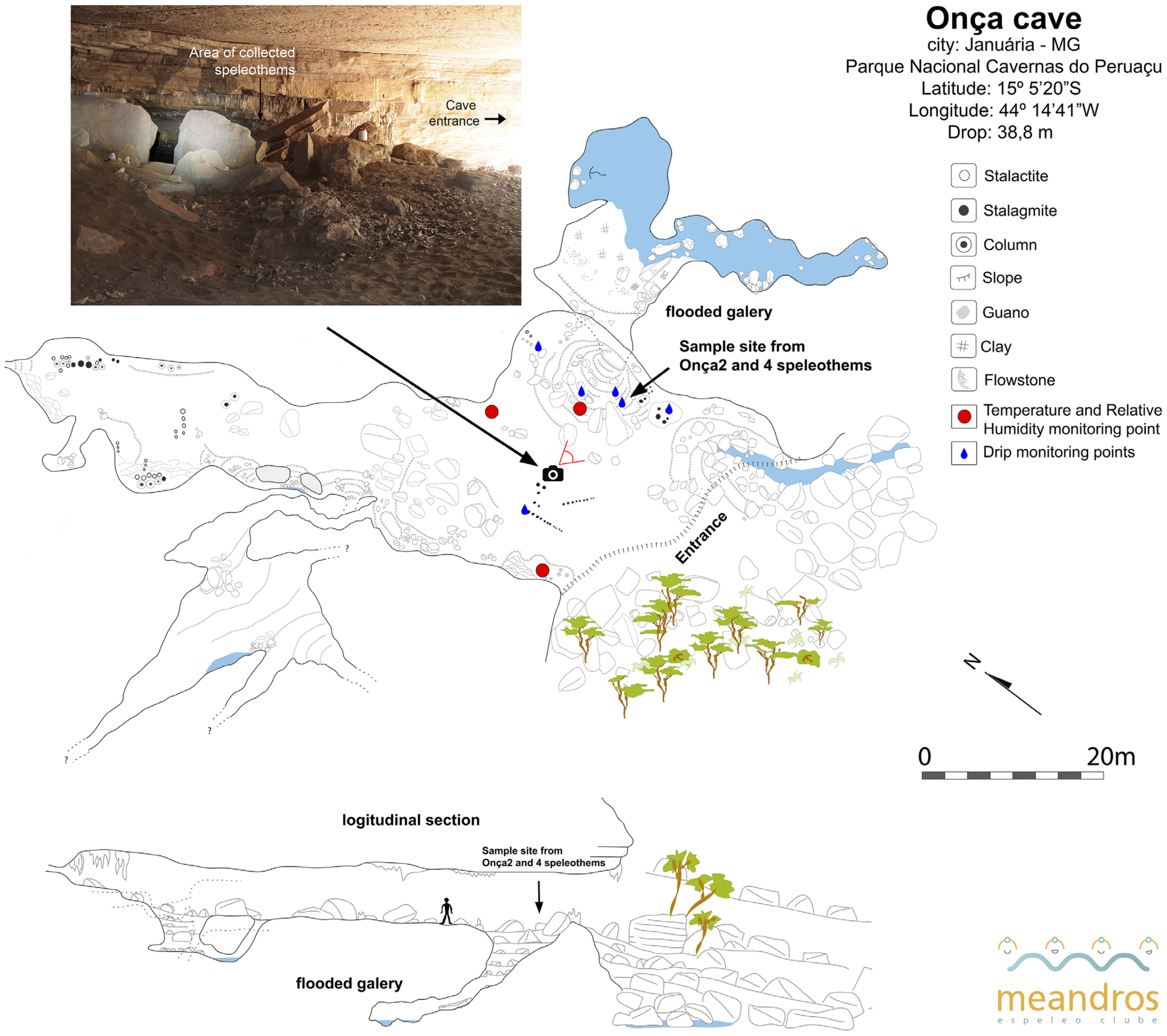

Figure S4 – Map of the Onça Cave, indicating the location of the speleothem sampling area.

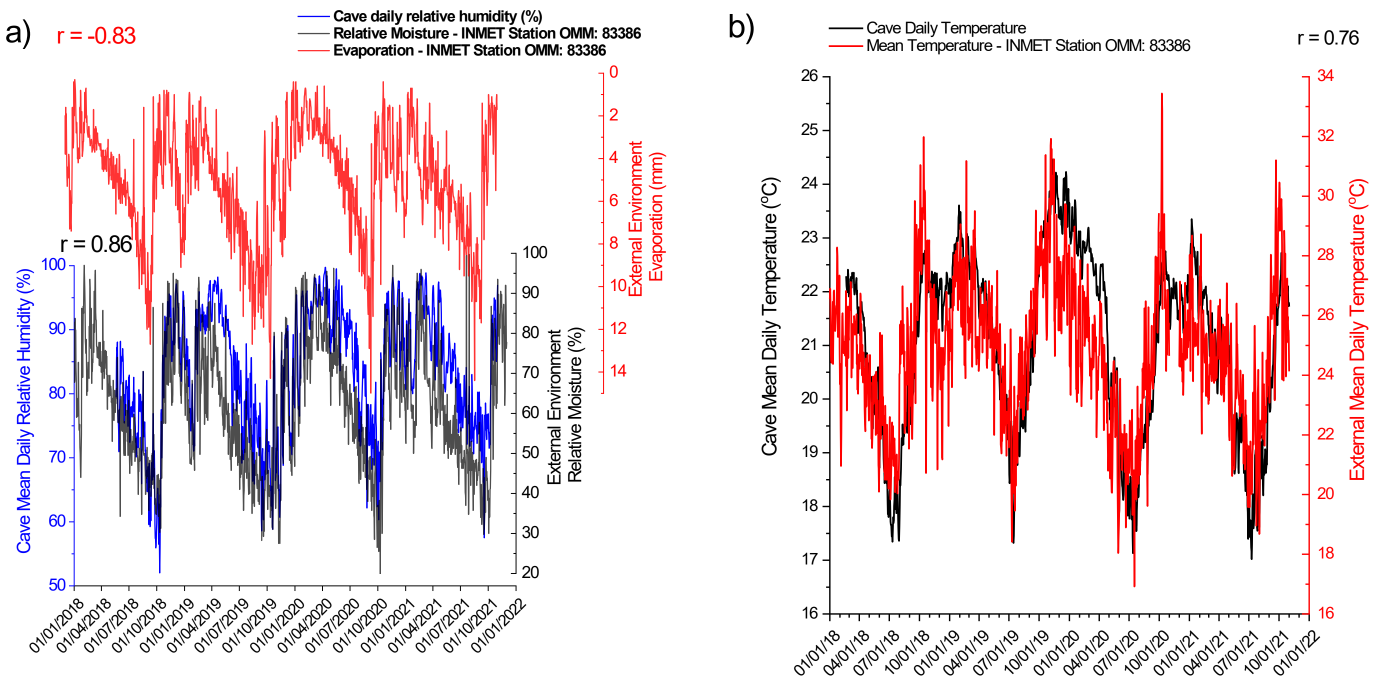

Figure S5 – Comparison between cave atmosphere relative humidity and temperature with the outside environment: a) relative humidity measured inside (blue) and outside the cave (black) and potential evaporation (red, inverted scale).; b) Onça Cave daily mean temperature (black) and the outside environment daily mean temperature. The exterior measurements were obtained from the local meteorological station from the *Instituto Nacional de Meteorologia* (INMET – OMM: 88336) at Januária City, located 40 km southwest of the cave. The potential evaporation was measured using a Piche evaporimeter. Note that scaling of relative humidity and temperature differs between left and right y-axis.

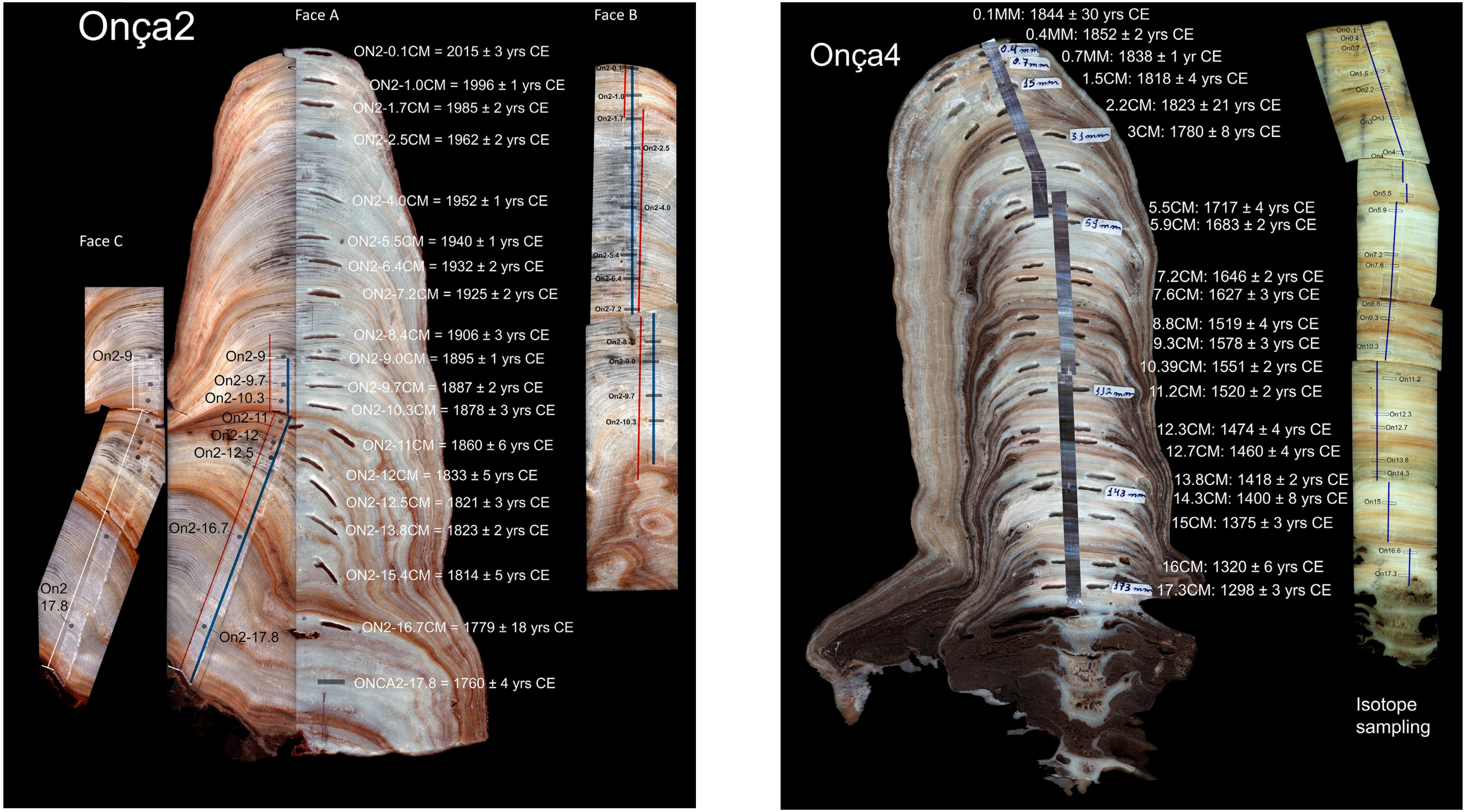

Figure S6 – Cross-section of speleothems Onça2 and Onça4 with isotope (blue) and trace element (red) profiles and U/Th ages indicated.

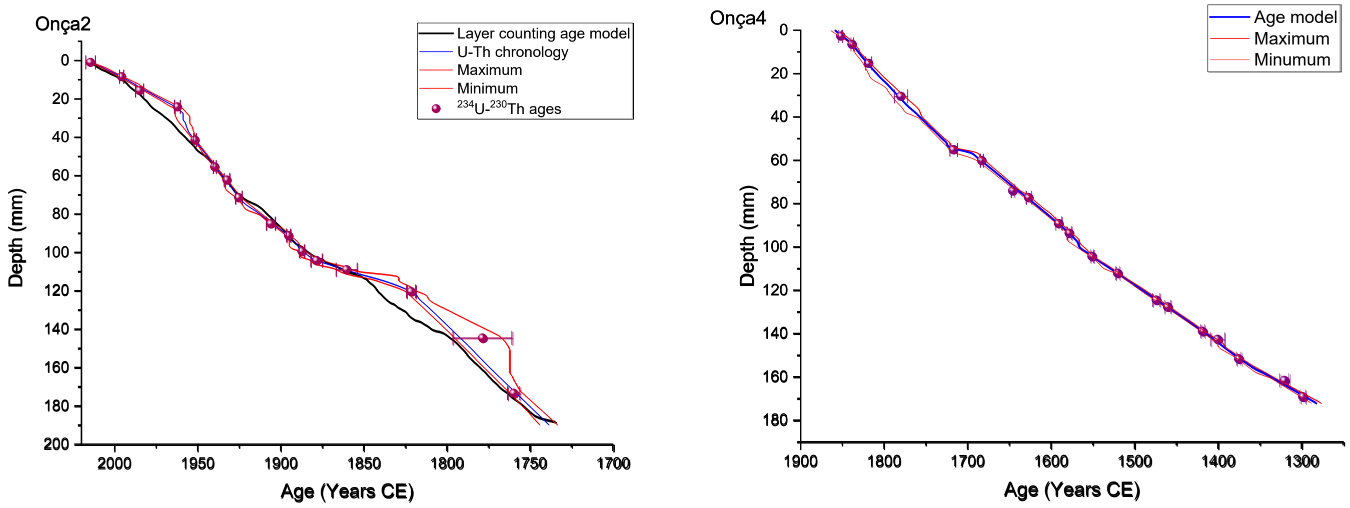

Figure S7 – Chronologies of Onça2 and Onça4 stalagmites: ^{234}U-^{230}Th based chronology (blue line) with linear interpolation age model using StalAge (Scholz and Hoffmann, 2011).

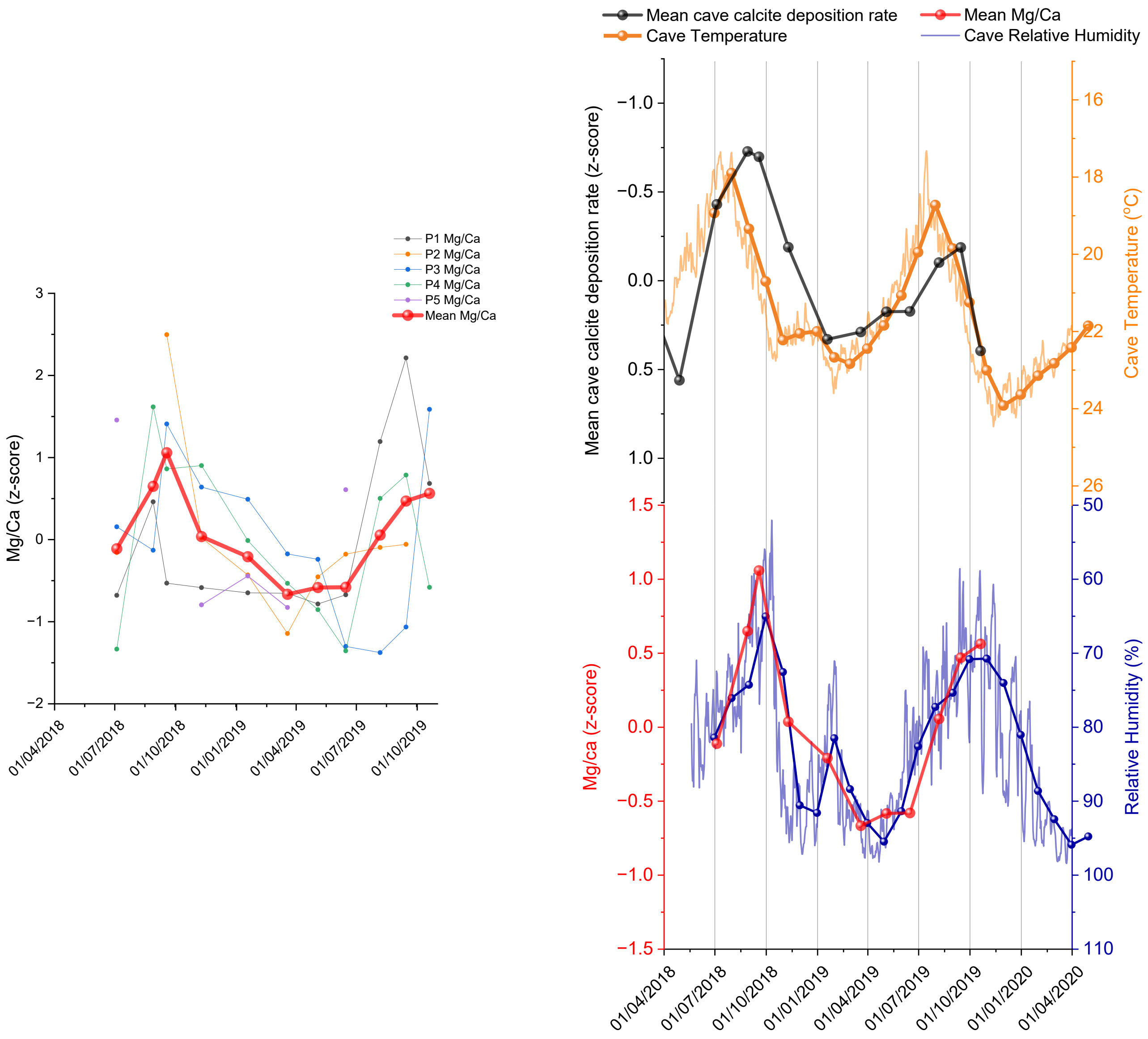

Figure S8 – Comparison between seasonal calcite Mg/Ca from monitoring experiment performed at Lapa da Onça and the cave relative humidity. Left: Time series of average monthly calcite Mg/Ca from Lapa da Onça cave experiment performed between June 2018 and October 2019 (red). The Mg/Ca of the dripping sites are presented as z-scored values. Right: comparison between the monthly mean Mg/Ca measured in the calcite deposition experiment (presented in the left panel) with cave relative humidity (blue lines), temperature (orange) and calcite deposition rate (black). Note that scales for mean calcite deposition, cave temperature and relative humidity are inverted.

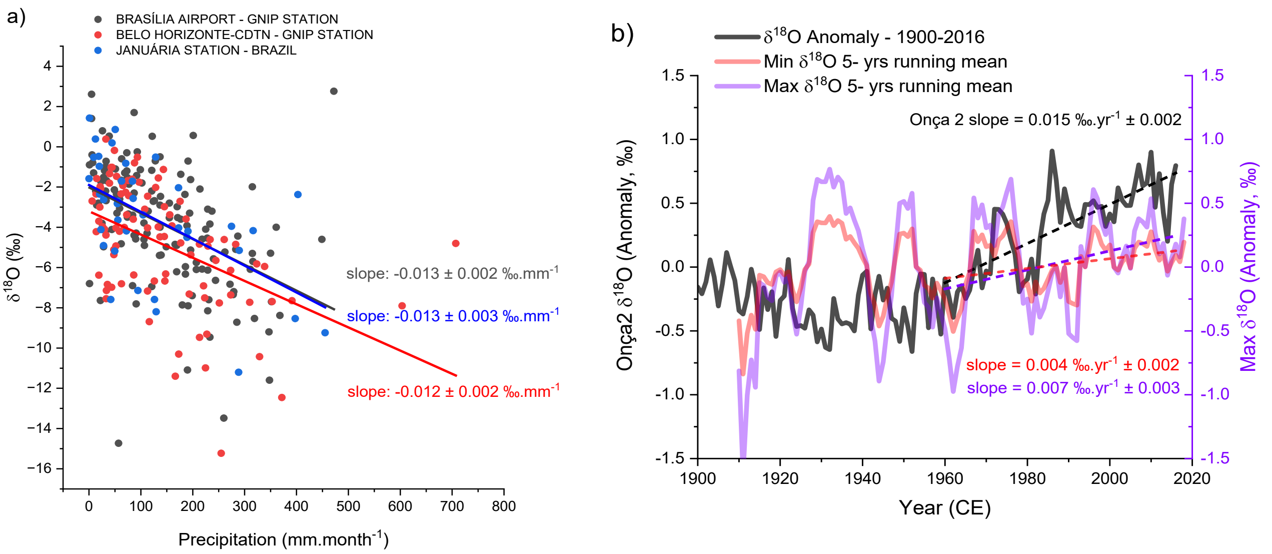

Figure S9 – a) Relationship between monthly $\delta^{18}O$ in rainfall and monthly precipitation. Black dots: data from Brasília GNIP Station (IAEA station: 1965-1987 - 15.85°S - 47.93°W), red dots: data from Belo Horizonte CDTN GNIP Station (IAEA station: 2008-2018 – 19.87°S; 43.97°W); blue dots: Januária-INMET Station (2011-2017 - 15.45°S; 44.37W°, this study); b) Comparison between Onça2 $\delta^{18}O$ record and estimated rainfall $\delta^{18}O$ with 5-year running mean. The estimated rainfall $\delta^{18}O$ was calculated using linear regressions derived from monthly mean precipitation and isotope rainfall monitoring data[2] (slope = -0.016 ± 0.002 ‰.mm^{-1}, n=21, r = -0,62, p < 0.001); Brasília GNIP Station (slope = -0.013 ± 0.002‰.mm^{-1}; n=135, n=-45, p<0.001); Belo Horizonte CDTN GNIP Station (slope = -0.012 ± 0.002 ‰.mm^{-1}; n=90, r =-0.49, p<0.001) and from Januária-INMET Station (slope = -0.013 ± 0.003 ‰.mm^{-1}; n=38, r =-0.55, p<0.001). The linear regressions obtained from the individual isotope monitoring experiments were used to estimate maximum and minimum slope between local rainfall (mm) and the $\delta^{18}O_{rainfall}$ (‰).

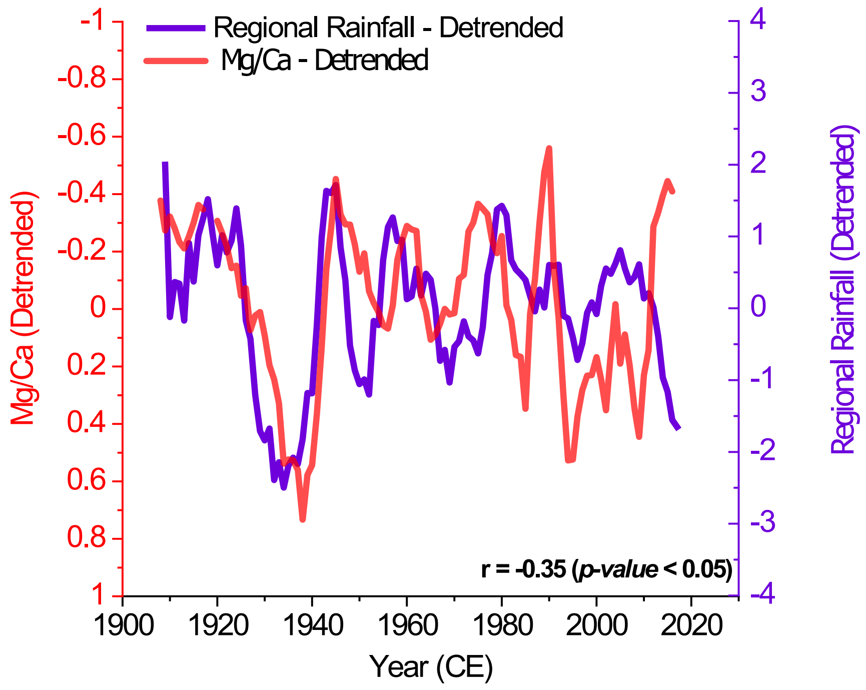

Figure S10 – Comparison between the detrended Mg/Ca (z-score) data and local rainfall time-series (z-score). The times series were detrended using a linear regression between 1908 to 2016. Note that the Mg/Ca scale is inverted.

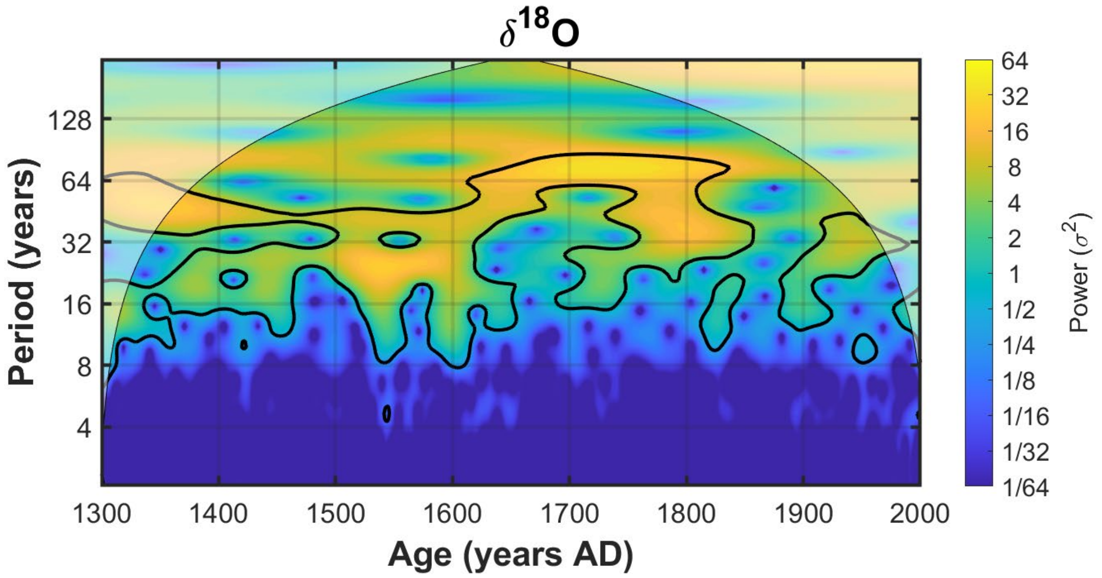

Figure S11 - Wavelet analysis performed with Onça2-4 δ^{18}O. Black lines indicate the 95% significance level and the cone of influence (region over which record length is sufficient to interpret results).

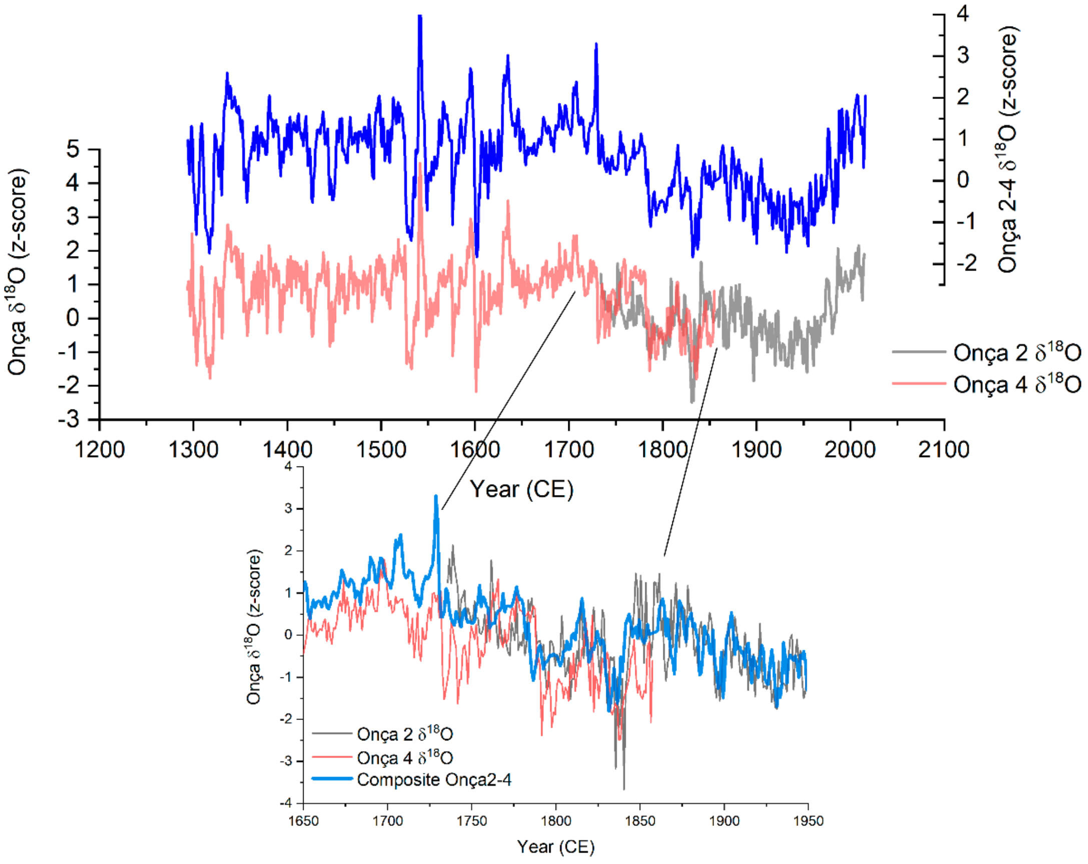

Figure S12 – Single composite curve of δ^{18}O from Onça speleothems using the iscam algorithm showing the overlapping period between 1723 and 1857 CE (bottom) (Fohlmeister, 2012).

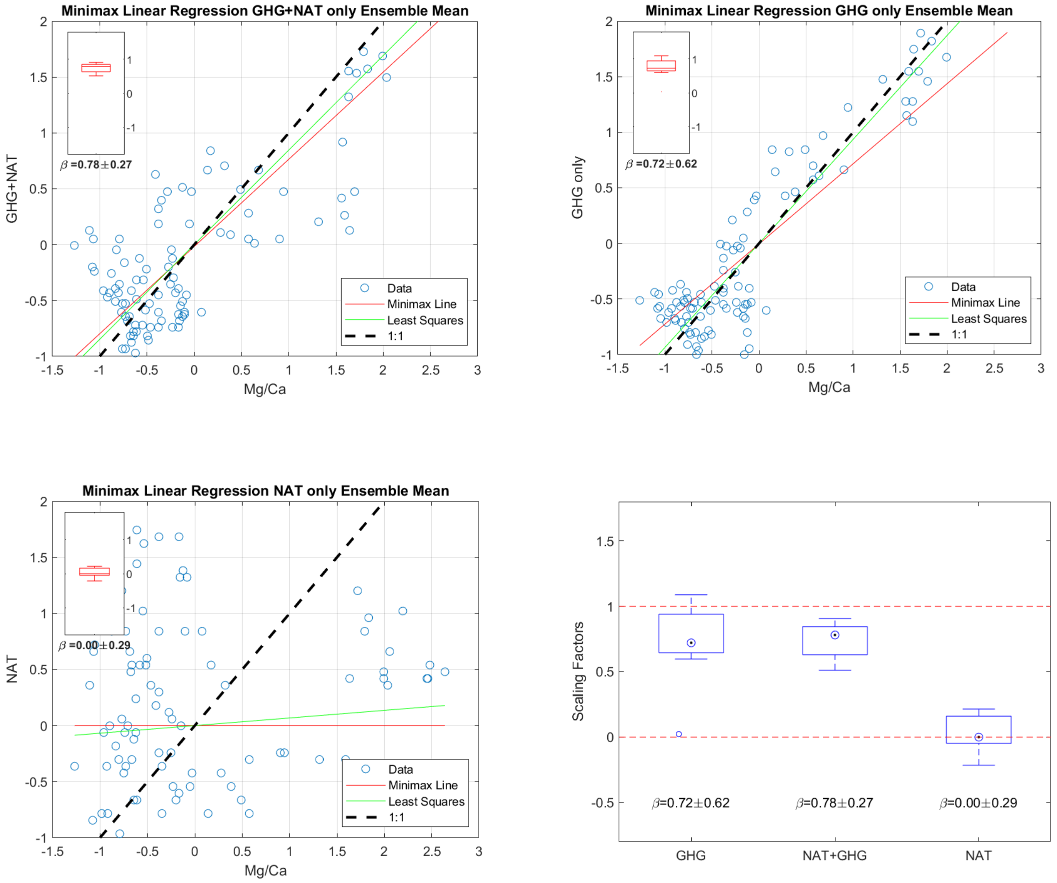

Figure S13 – Regression lines with their corresponding scaling factors (β-coefficient) from Mg/Ca vs. PET ensemble medians for NAT, GHG+NAT and GHG experiment. The whisker plot shows the β-coefficient obtained from 20 Monte Carlo simulations estimated based on the regression between proxy and P-PET from each ensemble member.

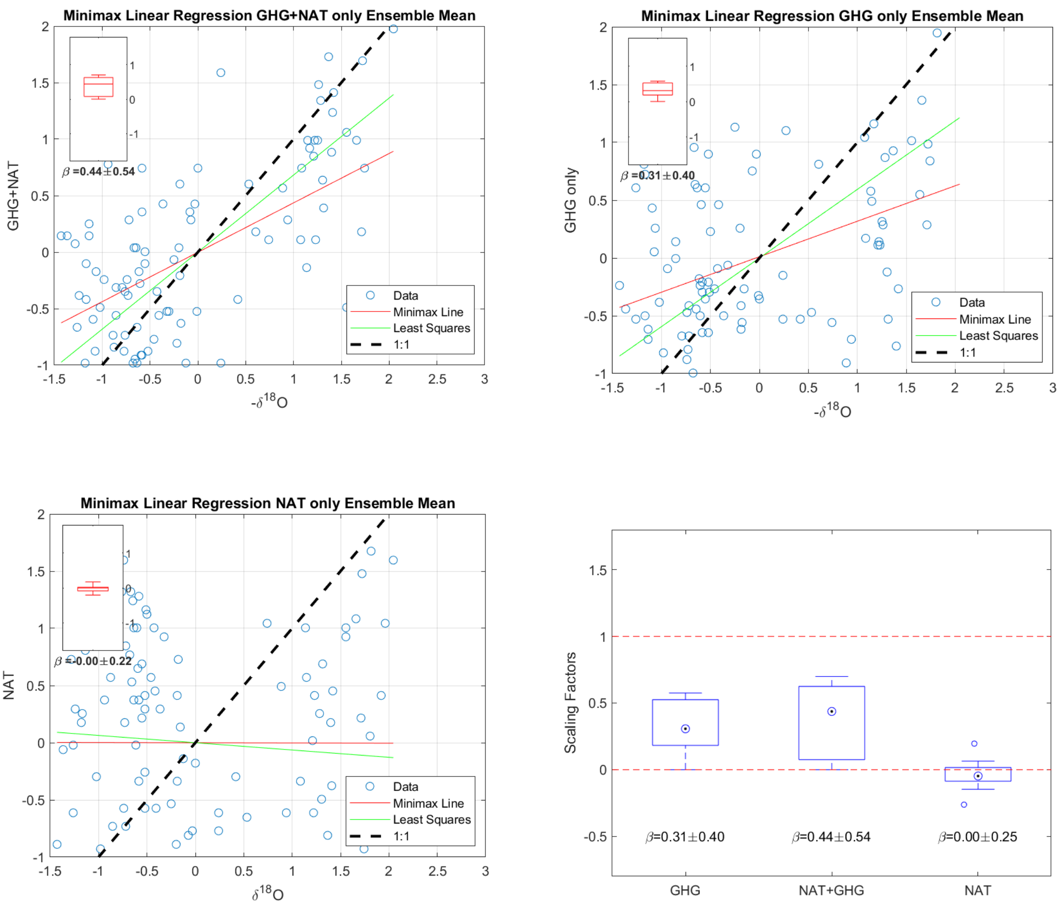

Figure S14 – Regression lines with their corresponding scaling factors (β-coefficient) from $\delta^{18}O$ vs P-PET ensemble medians for NAT, GHG+NAT and GHG experiment. The whisker plot shows the β-coefficient obtained from 20 Monte Carlo simulations estimated based on the regression between proxy and P-PET from each ensemble member.

Table S1 – List of stations used in this study

Temperature data

Station	Latitude	Longitude
Januária (OMM: 83386)	15°26'53.22"S	44°21'58.08"W
Montes Claros (OMM: 83437)	16°40'48.00"S	43°49'48.00"W
Pirapora (OMM: 83483)	17°21'0.00"S	44°54'36.00"W
Paracatu (OMM: 83479)	17°14'24.00"S	46°52'48.00"W
Salinas (OMM: 83441)	16° 9'36.00"S	42°17'60.00"W
Araçuaí (OMM: 83442)	16°49'48.00"S	42° 2'60.00"W
Espinosa (OMM: 83338)	14°54'36.00"S	42°47'60.00"W
Monte Azul (OMM-83388)	15° 9'36.00"S	42°51'36.00"W
Janúba (OMM: 83395)	15°48'0.00"S	43°17'24.00"W
Mocambinho (OMM: 83389)	15° 4'48.00"S	44° 0'36.00"W
Bom Jesus da Lapa (OMM: 83288)	13°15'36.00"S	43°24'36.00"W
Caetite (OMM: 83339)	14°35'60.00"S	42°28'48.00"W
Correntina (OMM: 83268)	13°19'48.00"S	44°36'36.00"W
Carinhanha (OMM: 83408)	14°16'48.00"S	43°45'36.00"W

Evaporation data

Station	Latitude	Longitude
Januária (OMM: 83386)	15°26'53.22"S	44°21'58.08"W
Montes Claros (OMM: 83437)	16°40'48.00"S	43°49'48.00"W
Pirapora (OMM: 83483)	17°21'0.00"S	44°54'36.00"W
Paracatu (OMM: 83479)	17°14'24.00"S	46°52'48.00"W
Salinas (OMM: 83441)	16° 9'36.00"S	42°17'60.00"W
Araçuaí (OMM: 83442)	16°49'48.00"S	42° 2'60.00"W
Bom Jesus da Lapa (OMM: 83288)	13°15'36.00"S	43°24'36.00"W
Caetite (OMM: 83339)	14°35'60.00"S	42°28'48.00"W
Carinhanha (OMM: 83408)	14°16'48.00"S	43°45'36.00"W
Correntina (OMM: 83268)	13°19'48.00"S	44°36'36.00"W
Espinosa (OMM: 83338)	14°54'36.00"S	42°47'60.00"W
Monte Azul (OMM-83388)	15° 9'36.00"S	42°51'36.00"W
Janaúba (OMM: 83395)	15°48'0.00"S	43°17'24.00"W
Mocambinho (OMM: 83389)	15° 4'48.00"S	44° 0'36.00"W

Pluviometric data

Stations	Latitude	Longitude
Araçuai (OMM: 83442)	16°49'48.00"S	42° 2'60.00"W
Bom Jesus da Lapa (OMM: 83288)	13°15'36.00"S	43°24'36.00"W
Caetite (OMM: 83339)	14°35'60.00"S	42°28'48.00"W
Carinhanha (OMM: 83408)	14°16'48.00"S	43°45'36.00"W
Correntinha (OMM: 83268)	13°19'48.00"S	44°36'36.00"W
Espinosa - MG (OMM: 83338)	14°54'36.00"S	42°47'60.00"W
Itacarambi (ANA: 01544024)	15° 4'59.88"S	44° 6'0.00"W
Januária (ANA: 1544007)	15°28'59.88"S	44°22'0.12"W
Janaúba (OMM: 83395)	15°48'0.00"S	43°17'24.00"W
Januária - MG (OMM: 83386)	15°26'53.22"S	44°21'58.08"W
Mocambinho - MG (OMM: 83389)	15° 4'48.00"S	44° 0'36.00"W

São Gonçalo (MONTAVANEA)- MG (ANA: 1444000)	14°18'48.96"S	44° 27' 37.08''W
Monte Azul (OMM-83388)	15° 9'36.00"S	42°51'36.00"W
Montes Claros (83437)	16°40'48.00"S	43°49'48.00"W
Usina Pandeiros - Montante (ANA: 01544032)	15° 28' 59.16"S	44° 46' 1.92''W
Pedras de Maria da Cruz (ANA: 01544010)	15° 36' 0.00"S	44° 24' 0.00''W
Pirapora (OMM: 83483)	17°21'0.00"S	44°54'36.00"W
Riacho da Cruz (ANA: 01544037)	15° 19' 4.08"S	44° 16' 0.12''W
Salinas (OMM: 83441)	16° 9'36.00"S	42°17'60.00"W
Varzelândia (ANA: 01544030)	15° 42' 15.12''S	44° 1' 42.96''W

Fluviometric data						
**Station**	**River**	**Latitude**	**Longitude**	**Start**	**End**	**Catchment area (km^2)**
ARROJADO (45770000)	Arrojado	13°27'10.08"S	44°34'8.04"W	1977	2019	5,540
FAZENDA BOM RETIRO (44540000)	Japoré	14°39'5.04"S	44° 5'33.00"W	1975	2011	624
CAPITÂNEA (45220000)	Coxá	14°25'24.96"S	44°28'59.16"W	1968	2016	2,380
MANGA (44500000)	São Franscisco	14°45'25.20"S	43°55'55.92"W	1933	2020	202,000
USINA DO PANDEIROS MONTANTE (44250000)	Pandeiros	15°28'59.16"S	44°46'4.80"W	1974	2016	3,230
SÃO GONÇALO (45131000)	Carinhanha	14°18'48.96"S	44°27'33.84"W	1947	2018	6,020
FAZENDA SANTA CRUZ (42546000)	Salobro	16° 8'4.92"S	46°44'44.16"W	1974	2018	553
BARRA DO ESCURO (43980002)	Urucuia	16°16'5.16"S	45°14'12.84"W	1963	2021	24,600
VILA URUCUIA (43670000)	Urucuia	16° 7'59.16"S	45°44'30.12"W	1963	2021	18,600
FAZENDA CONCEIÇÃO (43675000)	Ribeirão da Conceição	16°25'44.04"S	45°44'25.08"W	1984	2018	2,300
CAPITÃO ENÉAS (44630000)	Verde Grande	16°20'26.16"S	43°46'59.16"W	1973	2018	3,570
PONTE DA BR-040 - PRATA (423650000)	Rio da Prata	17°39'42.84"S	46°21'18.00"W	1973	2018	3,350
PORTO DOS POÇÕES (42600000)	Rio Preto	16°50'22.92"S	46°21'25.92"W	1957	2020	9,370
FAZENDA PORTO ALEGRE (45170001)	Itaguari	14°15'51.12"S	44°31'21.00"W	1964	2021	5,850

Table S2 – U-Th ages

^{230}Th dating results. The listed error is 2σ.

Sample Number	Depth mm	^{238}U (ppb)		^{232}Th (ppt)		^{230}Th / ^{232}Th (atomic x10^{-6})		δ^{234}U* (measured)		^{230}Th / ^{238}U (activity)		^{230}Th Age (yr) (uncorrected)		^{230}Th Age (yr) (corrected)		δ^{234}U$_{Initial}$** (corrected)		^{230}Th Age (yr BP)*** (corrected)		^{230}Th Age (CE) (corrected)	
**Onça 2**																					
**ON2-0.1CM**	**1 mm**	6254	±10	1714	±34	7	±1	1146	±2	0.0001	±0.0000	6	±1	**2**	**±3**	1146	±2	**-65**	**±3**	**2015**	±3
**ON2-1.0CM**	**10 mm**	6231	±24	672	±14	71	±2	1142	±4	0.0005	±0.0000	24	±1	**22**	**±1**	1143	±4	**-46**	**±1**	**1996**	±1
**ON2-1.7CM**	**17 mm**	4843	±19	1145	±23	50	±2	1152	±6	0.0007	±0.0000	36	±1	**33**	**±2**	1153	±6	**-35**	**±2**	**1985**	±2
**ON2-2.5CM**	**25 mm**	5136	±11	689	±14	139	±4	1148	±2	0.0011	±0.0000	58	±1	**56**	**±2**	1148	±2	**-12**	**±2**	**1962**	±2
**ON2-4.0CM**	**40 mm**	5590	±19	138	±3	878	±21	1149	±4	0.0013	±0.0000	67	±1	**66**	**±1**	1149	±4	**-2**	**±1**	**1952**	±1
**ON2-5.5CM**	**55 mm**	6507	±15	161	±4	1016	±24	1148	±3	0.0015	±0.0000	78	±1	**77**	**±1**	1148	±3	**10**	**±1**	**1940**	±1
**ON2-6.4CM**	**64 mm**	6072	±19	818	±17	211	±5	1153	±7	0.0017	±0.0000	87	±1	**86**	**±2**	1153	±7	**18**	**±2**	**1932**	±2
**ON2-7.2CM**	**72 mm**	5263	±10	872	±18	182	±4	1098	±2	0.0018	±0.0000	95	±1	**93**	**±2**	1098	±2	**25**	**±2**	**1925**	±2
**ON2-8.4CM**	**84 mm**	5909	±24	1526	±31	145	±3	1146	±4	0.0023	±0.0000	116	±1	**112**	**±3**	1146	±4	**44**	**±3**	**1906**	±3
**ON2-9.0CM**	**90 mm**	5635	±11	472	±10	477	±10	1150	±2	0.0024	±0.0000	123	±1	**122**	**±1**	1151	±2	**55**	**±1**	**1895**	±1
**ON2-9.7CM**	**97 mm**	5494	±18	637	±13	371	±8	1150	±4	0.0026	±0.0000	132	±1	**131**	**±2**	1150	±4	**63**	**±2**	**1887**	±2
**ON2-10.3CM**	**103 mm**	4656	±13	1560	±32	140	±3	1147	±3	0.0028	±0.0000	144	±1	**140**	**±3**	1147	±3	**72**	**±3**	**1878**	±3
**ON2-11.0CM**	**110 mm**	3778	±9	2369	±48	86	±2	1146	±3	0.0033	±0.0000	166	±2	**158**	**±6**	1146	±3	**90**	**±6**	**1860**	±6
**ON2-12.0CM**	**120 mm**	4264	±7	2356	±47	112	±2	1150	±2	0.0038	±0.0000	191	±1	**184**	**±5**	1150	±2	**117**	**±5**	**1833**	±5
**ON2-12.5CM**	**125 mm**	4705	±12	1153	±23	265	±6	1152	±3	0.0039	±0.0000	200	±1	**197**	**±3**	1152	±3	**129**	**±3**	**1821**	±3
**ON2-13.8CM**	**138 mm**	5599	±12	880	±18	407	±9	1150	±2	0.0039	±0.0000	197	±1	**195**	**±2**	1150	±2	**127**	**±2**	**1823**	±2
**ON2-15.4CM**	**154 mm**	4728	±12	2520	±51	129	±3	1149	±3	0.0042	±0.0000	212	±2	**204**	**±5**	1150	±3	**136**	**±5**	**1814**	±5
**ON2-16.7CM**	**167 mm**	4171	±7	7591	±152	46	±1	1122	±2	0.0051	±0.0000	263	±2	**238**	**±18**	1122	±2	**171**	**±18**	**1779**	±18
**ONCA2-17.8**	**178 mm**	4031	±12	1161	±24	296	±7	1151	±3	0.0052	±0.0000	262	±2	**258**	**±4**	1152	±3	**190**	**±4**	**1760**	±4
**Onça 4**																					
**ON4-0.1MM**	**1 mm**	4500	±9	13197	±265	23	±1	1033	±2	0.0040	±0.000043	215	±2	**173**	**±30**	**1033**	±2	**106**	**±30**	**1844**	±30
**ON4-0.4MM**	**4 mm**	6435	±21	1320	±27	252	±5	1038	±4	0.0031	±0.000019	168	±1	**165**	**±2**	**1039**	**±4**	**98**	**±2**	**1852**	±2
**ON4-0.7MM**	**7 mm**	6835	±15	473	±10	798	±17	1036	±3	0.0034	±0.000023	180	±1	**179**	**±1**	**1036**	**±3**	**112**	**±1**	**1838**	±1
**ON4-1.5CM**	**15 mm**	5436	±11	1796	±36	190	±4	1040	±2	0.0038	±0.000022	203	±1	**199**	**±4**	**1041**	**±2**	**132**	**±4**	**1818**	±4
**ON4-2.2CM**	**22 mm**	6344	±13	13031	±262	34	±1	1040	±2	0.00419	±0.000027	224.0	±1	**195**	**±21**	**1041**	**±2**	**127**	**±21**	**1823**	±21
**ON4-3CM**	**30 mm**	5078	±9	3856	±77	100	±2	1031	±2	0.0046	±0.000041	248	±2	**237**	**±8**	**1032**	**±2**	**170**	**±8**	**1780**	±8
**ON4-4.CM**	**41 mm**	8825	±21	68	±2	156170	±4487	1022	±3	0.0734	±0.000181	4018	±11	**4018**	**±11**	**1034**	**±3**	**3950**	**±11**	**-2000**	±11
**ON4-5.5CM**	**55 mm**	5415	±12	2197	±44	230	±5	1017	±3	0.0057	±0.000026	307	±1	**301**	**±4**	**1018**	**±3**	**233**	**±4**	**1717**	±4
**ON4-5.9CM**	**59 mm**	6407	±13	571	±12	1137	±24	1003	±2	0.0061	±0.000028	335	±2	**334**	**±2**	**1004**	**±2**	**267**	**±2**	**1683**	±2

**ON4-7.2CM**	**72 mm**	5879	±24	332	±7	1974	±41	982	±3	0.0068	±0.000034	373	±2	**372**	**±2**	**983**	**±3**	**304**	**±2**	**1646**	**±2**
**ON4-7.6CM**	**76 mm**	6240	±14	1541	±31	480	±10	988	±3	0.0072	±0.000030	395	±2	**391**	**±3**	**989**	**±3**	**323**	**±3**	**1627**	**±3**
**ON4-8.8CM**	**88 mm**	6647	±14	2293	±46	379	±8	1006	±2	0.0079	±0.000029	432	±2	**427**	**±4**	**1007**	**±2**	**359**	**±4**	**1591**	**±4**
**ON4-9.3CM**	**93 mm**	5665	±10	651	±13	1146	±24	975	±2	0.0080	±0.000046	442	±3	**440**	**±3**	**976**	**±2**	**372**	**±3**	**1578**	**±3**
**ON4-10.39CM**	**103 mm**	7327	±16	570	±12	1820	±38	1002	±2	0.0086	±0.000032	468	±2	**467**	**±2**	**1004**	**±2**	**399**	**±2**	**1551**	**±2**
**ON4-11.2CM**	**112 mm**	6256	±12	438	±9	2166	±45	1017	±2	0.0092	±0.000035	498	±2	**497**	**±2**	**1019**	**±2**	**430**	**±2**	**1520**	**±2**
**ON4-12.3CM**	**123 mm**	4485	±8	1706	±34	455	±9	1087	±2	0.0105	±0.000038	549	±2	**544**	**±4**	**1089**	**±2**	**476**	**±4**	**1474**	**±4**
**ON4-12.7CM**	**127 mm**	4387	±7	1149	±23	626	±13	934	±2	0.0099	±0.000047	562	±3	**558**	**±4**	**936**	**±2**	**490**	**±4**	**1460**	**±4**
**ON4-13.8CM**	**138 mm**	5392	±11	201	±4	5452	±119	1245	±3	0.0123	±0.000040	600	±2	**600**	**±2**	**1247**	**±3**	**532**	**±2**	**1418**	**±2**
**ON4-14.3CM**	**143 mm**	4266	±8	3633	±73	249	±5	1243	±3	0.0129	±0.000048	628	±2	**617**	**±8**	**1245**	**±3**	**550**	**±8**	**1400**	**±8**
**ON4-15.0CM**	**150 mm**	4596	±9	625	±13	1599	±33	1236	±3	0.0132	±0.000047	645	±2	**643**	**±3**	**1238**	**±3**	**575**	**±3**	**1375**	**±3**
**ON4-16.6CM**	**166 mm**	3601	±8	1907	±38	460	±9	1292	±3	0.0148	±0.000055	704	±3	**698**	**±6**	**1295**	**±3**	**630**	**±6**	**1320**	**±6**
**ON4-17.3CM**	**173 mm**	3854	±6	411	±9	2333	±50	1289	±3	0.0151	±0.000061	720	±3	**719**	**±3**	**1292**	**±3**	**652**	**±3**	**1298**	**±3**

U decay constants: $\lambda_{238} = 1.55125x10^{-10}$ (Jaffey et al., 1971) and $\lambda_{234} = 2.82206x10^{-6}$ (Cheng et al., 2013). Th decay constant: $\lambda_{230} = 9.1705x10^{-6}$ (Cheng et al., 2013). *$\delta^{234}U = ([^{234}U/^{238}U]_{activity} - 1)x1000$.
** $\delta^{234}U_{initial}$ was calculated based on ^{230}Th age (T), i.e., $\delta^{234}U_{initial} = \delta^{234}U_{measured}$ x $e^{\lambda 234xT}$.
Corrected ^{230}Th ages assume the initial $^{230}Th/^{232}Th$ atomic ratio of 4.4 ± 2.2 $x10^{-6}$. Those are the values for a material at secular equilibrium, with the bulk earth $^{232}Th/^{238}U$ value of 3.8. The errors are arbitrarily assumed to be 50%.
***B.P. stands for "Before Present" where the "Present" is defined as the year 1950 CE

Table S3 – Earth system models from CMIP6 used in the analysis.

Model	Institute	Resolution	Reference
**CanESM5**: Canadian Earth System Model version 5	Canadian Centre for Climate Modelling and Analysis (CCCma)	2.8º (atmosphere), 1.0º (ocean)	Swart et al., 2019
**CESM2**: Community Earth System Model Version 2 (CESM2)	National Center for Atmospheric Research (NCAR)	1.25° lon x 0.9° lat (atmosphere), 0.27-0.64º (ocean)	Danabasoglu et al., 2020
**GFDL-ESM4**: Earth System Model version 4	Geophysical Fluid Dynamics Laboratory (GFDL)	1º (atmosphere), 0.5º (ocean.)	Dune et al., 2020
**GISS-E2-1-G**: Goddard Institute for Space Studies (GISS) E2.1 model	National Aeronautics and Space Administration (NASA)	2 º ×2.5 º (atmosphere), 1 º ×1.25 º (ocean)	Miller et al., 2020
**HadGEM3-GC31-LL**: Hadley Centre Global Environment Model 3	Met Office Hadley Centre	~135 km (atmosphere), 1° (ocean)	Kuhlbrodt et al., 2018
**IPSL-CM6A-LR**: IPSL climate model version 6	Institut Pierre-Simon Laplace (IPSL)	96 × 95 points in lon-lat (atmosphere), 2º (ocean)	Boucher et al., 2019
**MIROC6**: Sixth version of the Model for Interdisciplinary Research on Climate	Center for Climate System Research (CCSR), the University of Tokyo, the Japan Agency for Marine-Earth Science and Technology (JAMSTEC), and the National Institute for Environmental Studies (NIES)	1.4º (atmosphere), 0.5-1º (ocean)	Tatebe et al., 2019
**MRI-ESM2-0**: Meteorological Research Institute - Earth System Model version 2.0	Japan Meteorological Agency	100 km for atmosphere and ocean components	Yukimoto et al., 2019

Table S4 – p-values of the t-test of regressions calculated in the Detection and Attribution analysis using the minmax algorithm[11].

Mg/Ca vs PET

Hypotheses	T*	ν*	p-value
**H_0: $\beta_{GHG\_only} = \beta_{nat\_GHG}$**	**-0.27**	**10**	**0.794**
H_0: $\beta_{GHG\_only} = \beta_{nat\_only}$	5.77	13	0.0001
H_0: $\beta_{nat\_GHG} = \beta_{nat\_only}$	10.17	12	>0.0001

Hypotheses	T*	ν*	p-value
H_0: $\beta_{GHG\_only} = 1$	3.83	8	0.005
H_0: $\beta_{nat\_GHG} = 1$	11.17	8	>0.0001
**H_0: $\beta_{nat\_only} = 0$**	**0.49**	**8**	**0.6384**

$\delta^{18}O$ vs P-PET

Hypotheses	T*	ν*	p-value
**H_0: $\beta_{GHG\_only} = \beta_{nat\_GHG}$**	**-0.44**	**15**	**0.6684**
H_0: $\beta_{GHG\_only} = \beta_{nat\_only}$	4.57	12	0.0006
H_0: $\beta_{nat\_GHG} = \beta_{nat\_only}$	4.45	11	0.0010

Hypotheses	T*	ν*	p-value
H_0: $\beta_{GHG\_only} = 1$	9.33	8	>0.0001
H_0: $\beta_{nat\_GHG} = 1$	7.19	8	0.0001
**H_0: $\beta_{nat\_only} = 0$**	**-0.56**	**8**	**0.5916**

***T is the value of the t-test and ν is degree of freedom calculated accordingly to the following equation:**

$$T = \frac{\overline{\beta_1} - \overline{\beta_2}}{\sqrt{\frac{S_1}{n_1} + \frac{S_2}{n_2}}} \qquad \nu = \frac{(A^2 - B^2)}{\frac{A^2}{(n_1 - 1)} + \frac{B^2}{(n_2 - 1)}} \qquad A = \frac{S_1^2}{n_1} \; and \; B = \frac{S_2^2}{n_2}$$

Supplementary References

1. Killick, R., Fearnhead, P. & Eckley, I. A. Optimal detection of changepoints with a linear computational cost. *J. Am. Stat. Assoc.* **107**, 1590–1598 (2012).

2. Moquet, J. S. *et al.* Calibration of speleothem $\delta^{18}O$ records against hydroclimate instrumental records in Central Brazil. *Glob. Planet. Change* **139**, (2016).

3. Swart, N. C. *et al.* The Canadian Earth System Model version 5 (CanESM5.0.3). *Geosci. Model Dev.* **12**, 4823–4873 (2019).

4. Danabasoglu, G. *et al.* The Community Earth System Model Version 2 (CESM2). *J. Adv. Model. Earth Syst.* **12**, 1–35 (2020).

5. Dunne, J. P. *et al.* The GFDL Earth System Model Version 4.1 (GFDL-ESM 4.1): Overall Coupled Model Description and Simulation Characteristics. *J. Adv. Model. Earth Syst.* **12**, 1–56 (2020).

6. Miller, R. L. *et al.* CMIP6 Historical Simulations (1850–2014) With GISS-E2.1. *J. Adv. Model. Earth Syst.* **13**, e2019MS002034 (2021).

7. Kuhlbrodt, T. *et al.* The Low-Resolution Version of HadGEM3 GC3.1: Development and Evaluation for Global Climate. *J. Adv. Model. Earth Syst.* **10**, 2865–2888 (2018).

8. Boucher, O. *et al.* Presentation and Evaluation of the IPSL-CM6A-LR Climate Model. *J. Adv. Model. Earth Syst.* **12**, (2020).

9. Tatebe, H. *et al.* Description and basic evaluation of simulated mean state, internal variability, and climate sensitivity in MIROC6. *Geosci. Model Dev.* **12**, 2727–2765 (2019).

10. Yukimoto, S. *et al.* The meteorological research institute Earth system model version 2.0, MRI-ESM2.0: Description and basic evaluation of the physical component. *J. Meteorol. Soc. Japan* **97**, 931–965 (2019).

11. Xu, Q. & Xuan, X. M. Nonlinear regression without i.i.d. assumption. *Probab. Uncertain. Quant. Risk* **4**, (2019).
