# Supplementary material for: Modern anthropogenic drought in Central Brazil unprecedented during last 700 years
Source: Nat Commun. 2024 Feb 26;15:1728. doi: 10.1038/s41467-024-45469-8 (PMC11258244; doi:10.1038/s41467-024-45469-8)
Supplement: Supplementary file 3 — Description of Additional Supplementary Files [file 41467_2024_45469_MOESM3_ESM.pdf]

Description of Additional Supplementary Files

File Name: Supplementary Data 1
Description: Isotopes and trace element records from Onça2 and Onça4 stalagmites.

File Name: Supplementary Data 2
Description: Regional precipitation, potential evapotranspiration; hydrologic balance and streamflow.

File Name: Supplementary Data 3
Description:
•Cave dripping isotope monitoring are presented in the in tab “Cave dripping isotope data”
•Modern calcite deposition and Mg/Ca ratios from farmed calcite generated in the cave monitoring are presented in the tab “Monitoring Mg_Ca and dep rate”.
•Cave atmospheric temperature and relative humidity are presented in the tab “Cave temp. and RH

File Name: Supplementary Data 4
Description: Local and regional rainfall isotope data.

File Name: Supplementary Data 5
Description: Surface temperature reconstruction from the Paleo Hydrodynamics Data Assimilation (PHYDA).

File Name: Supplementary Data 6
Description: Potential evapotranspiration and hydrologic balance (P-PET) calculated from CMIP6 experiments GHG+NAT, GHG and NAT are presented in the tabs “CMIP6_GHG+NAT”, CMIP6_GHG and “CMIP6_NAT” respectively.
