# Supplementary figures and images for: Modern anthropogenic drought in Central Brazil unprecedented during last 700 years
Source: Nat Commun. 2024 Feb 26;15:1728. doi: 10.1038/s41467-024-45469-8 (PMC11258244; doi:10.1038/s41467-024-45469-8)

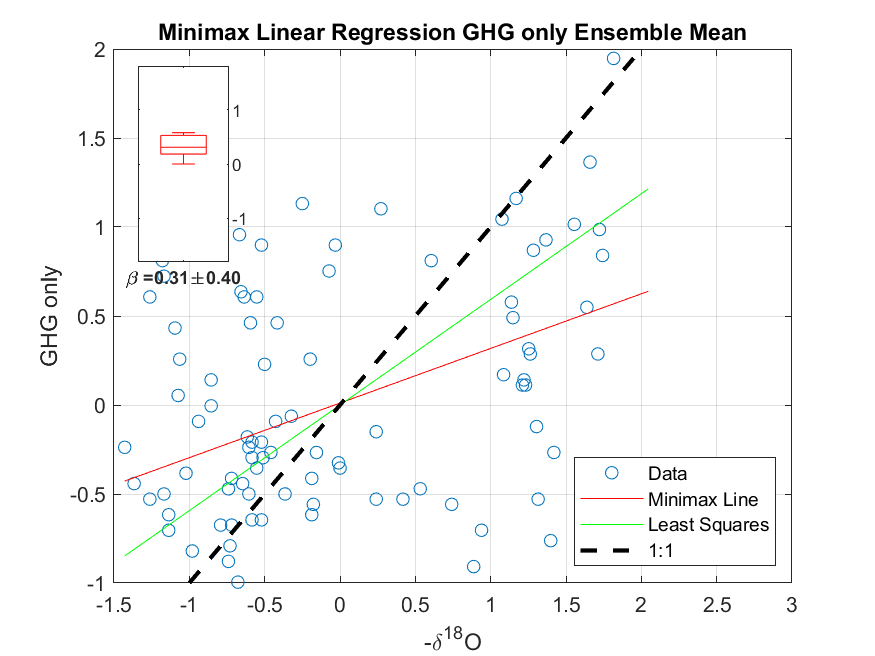

Supplement: Supplementary file 10 — Source Data [file 41467_2024_45469_MOESM10_ESM.zip › Supplementary Code 1/D2_1_run2.png]

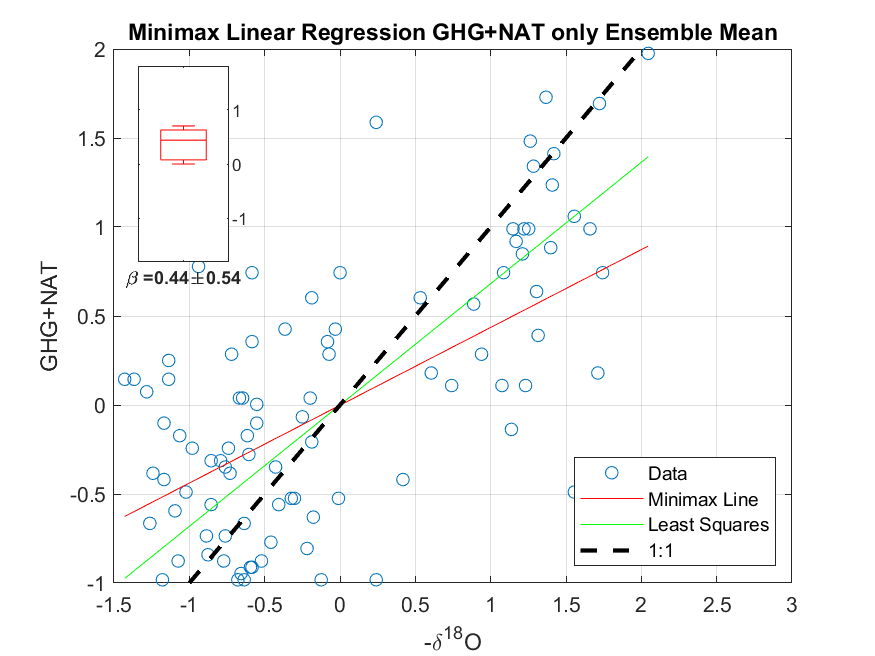

Supplement: Supplementary file 10 — Source Data [file 41467_2024_45469_MOESM10_ESM.zip › Supplementary Code 1/D2_2_run2.png]

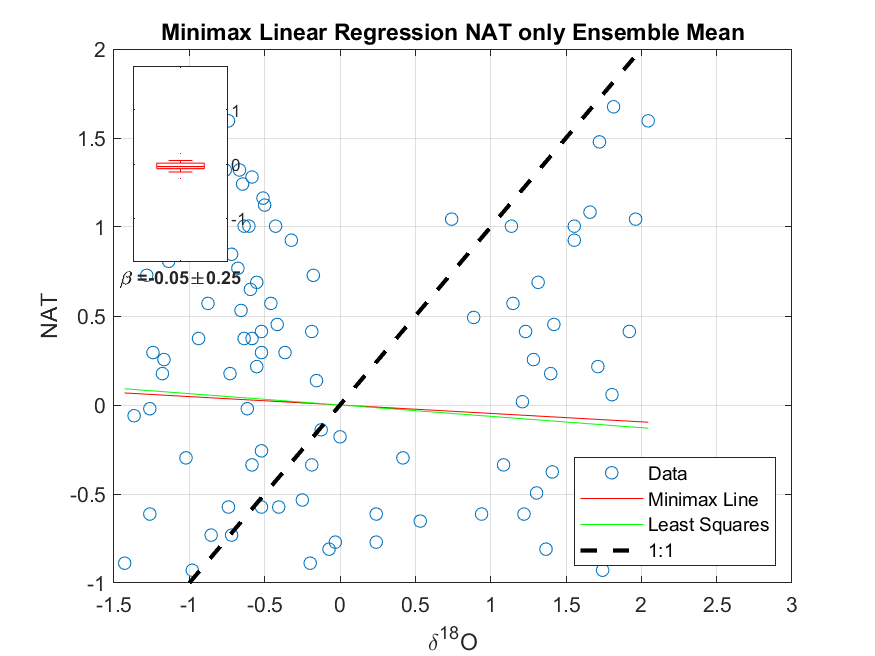

Supplement: Supplementary file 10 — Source Data [file 41467_2024_45469_MOESM10_ESM.zip › Supplementary Code 1/D2_3_2.png]

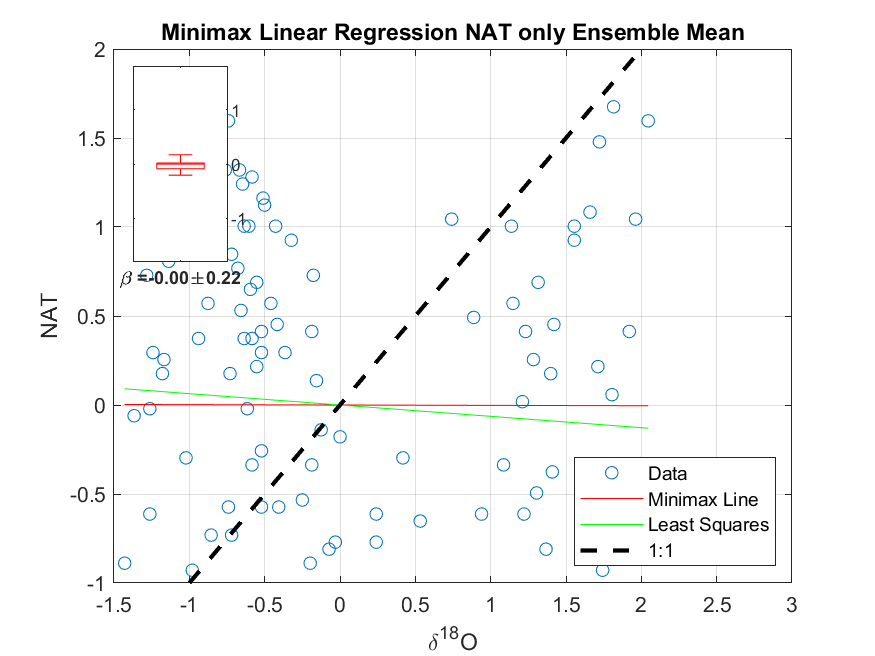

Supplement: Supplementary file 10 — Source Data [file 41467_2024_45469_MOESM10_ESM.zip › Supplementary Code 1/D2_3_run2.png]

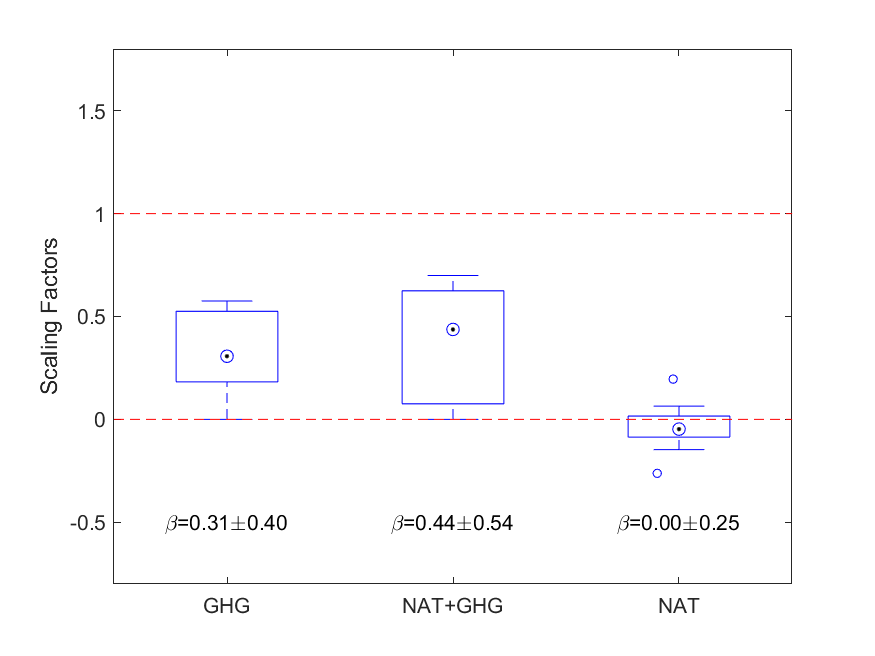

Supplement: Supplementary file 10 — Source Data [file 41467_2024_45469_MOESM10_ESM.zip › Supplementary Code 1/D2_Boxplot_2.png]

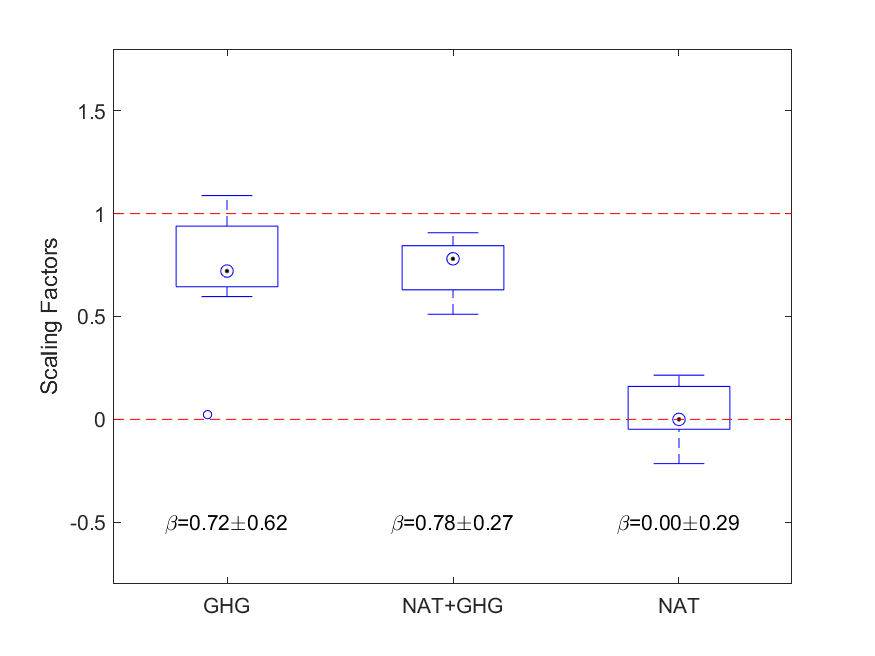

Supplement: Supplementary file 10 — Source Data [file 41467_2024_45469_MOESM10_ESM.zip › Supplementary Code 1/D2_Boxplot_run2.png]

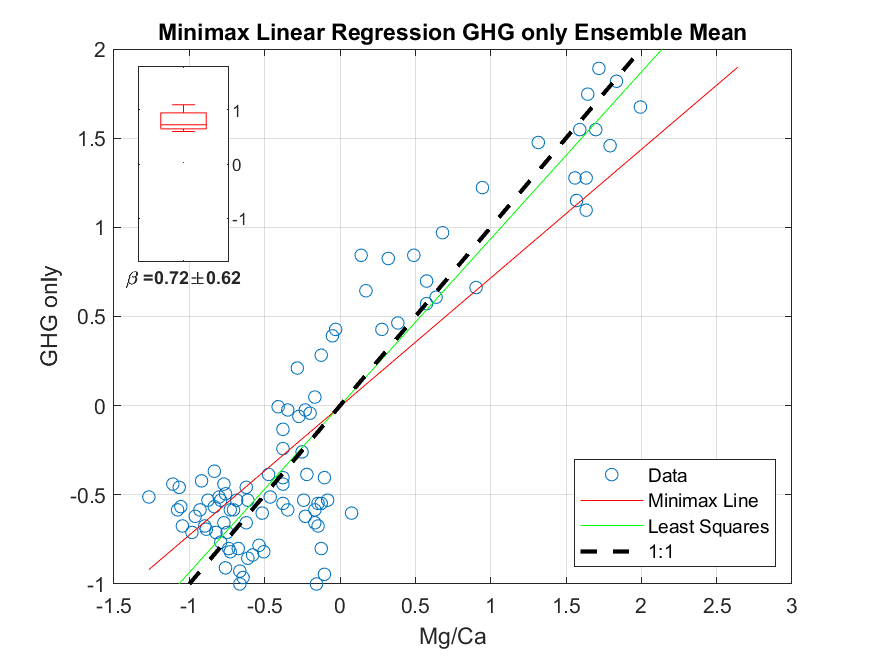

Supplement: Supplementary file 10 — Source Data [file 41467_2024_45469_MOESM10_ESM.zip › Supplementary Code 1/M2_1_run2.png]

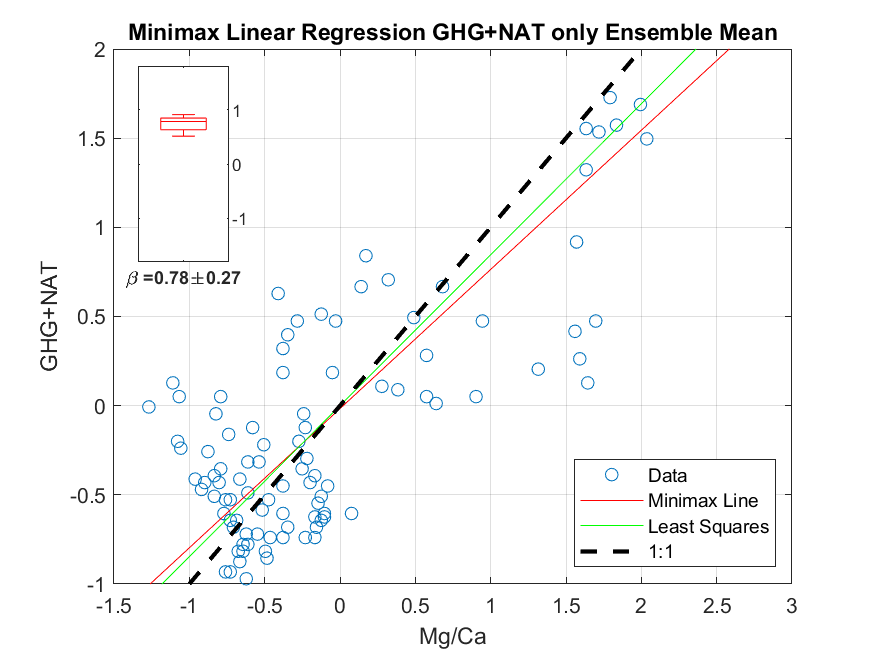

Supplement: Supplementary file 10 — Source Data [file 41467_2024_45469_MOESM10_ESM.zip › Supplementary Code 1/M2_2_run2.png]

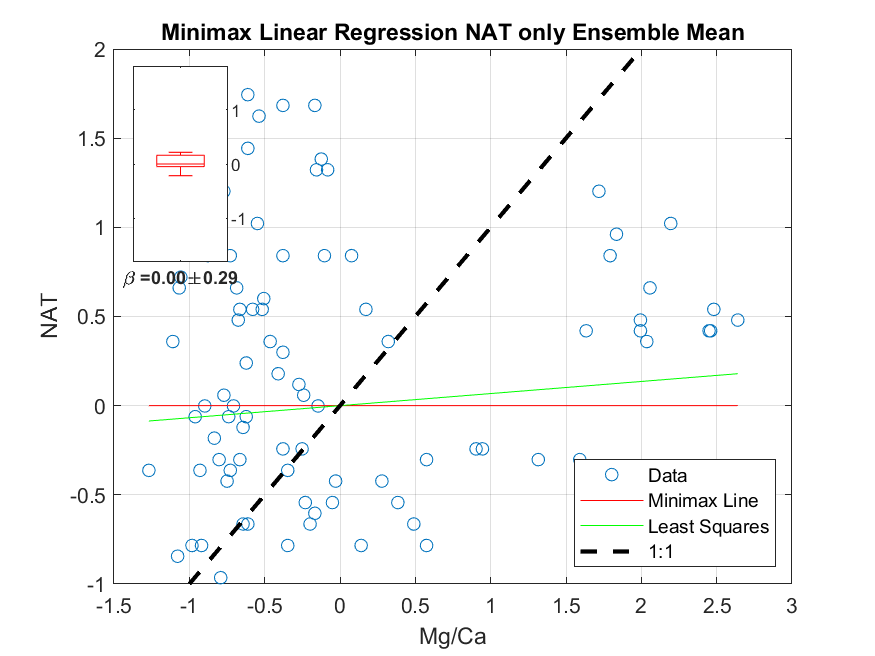

Supplement: Supplementary file 10 — Source Data [file 41467_2024_45469_MOESM10_ESM.zip › Supplementary Code 1/M2_3_run2.png]
